# Supplementary material for: Development and initial testing of a brief, generic self-reported disability questionnaire: The Universal Disability Index
Source: PLoS One. 2024 May 8;19(5):e0303102. doi: 10.1371/journal.pone.0303102 (PMC11078367; doi:10.1371/journal.pone.0303102)
Supplement: S1 Table — (PDF) [file pone.0303102.s001.pdf]

Table S1. UDI question wording

Activity	Question wording	Response options
Walking	How much have you been able to walk during the past 2 weeks?	0 I have been able to walk more than 1 mile at a time<br>1 I have been able to walk up to 1 mile at a time<br>2 I have been able to walk up to 1/4 of a mile at a time<br>3 I have been able to walk up to 100 yards at a time<br>4 I have only been able to walk using a stick, crutches or handrails<br>5 I have not been able to walk at all (e.g. I had to use a wheelchair or crawl to the toilet)
Standing	How much have you been able to stand during the past 2 weeks?	0 I have been able to stand as long as I want<br>1 I have been able to stand as long as I want but it was difficult to do so<br>2 I have been able to stand for no more than 1 hour at a time<br>3 I have been able to stand for no more than 30 minutes at a time<br>4 I have been able to stand for no more than 10 minutes at a time<br>5 I have not been able to stand at all
Sitting	How much have you been able to sit during the past 2 weeks?	0 I have been able to sit as long as I want in any chair<br>1 I have only been able to sit as long as I want in my favourite chair<br>2 I have been able to sit for up to 1 hour at a time

		3 I have been able to sit for up to 30 minutes at a time<br>4 I have been able to sit for up to 10 minutes at a time<br>5 I have not been able to sit at all
Lifting and carrying	How much have you been able to lift or carry objects during the past 2 weeks?	0 I have been able to lift or carry heavy objects without any difficulty at all<br>1 I have been able to lift or carry heavy objects but it was difficult to do so<br>2 I have only been able to lift or carry heavy objects if they were conveniently positioned (e.g. on a table)<br>3 I have only been able to lift or carry light to medium weight objects if they were conveniently positioned<br>4 I have only been able to lift or carry very light objects<br>5 I have not been able to lift or carry anything at all
Work and daily routine	How much have you been able to work (including your daily routine, household activities, preparing food, etc.) during the past 2 weeks?	0 I have been able to do as much work as I want to<br>1 I have been able to do all of my usual work, but no more<br>2 I have been able to do most of my usual work, but no more<br>3 I have not been able to do my usual work<br>4 I could hardly do any work at all<br>5 I have been unable to do any work at all
Personal care (washing, dressing, etc.)	How much have you been able to wash and dress during the past 2 weeks?	0 I have been able to wash and dress myself normally

		1 I have been able to wash and dress myself but this has been difficult<br>2 I have been able to wash and dress myself but had to be slow and careful when doing so<br>3 I managed to wash and dress myself most of the time but needed some help<br>4 I managed to wash and dress myself some of the time but needed lots of help<br>5 I could not wash or dress myself at all and either relied upon help or stayed in bed
Sleeping	How much have you been able to sleep during the past 2 weeks?	0 My sleep was never disturbed<br>1 My sleep was occasionally disturbed (less than 1 hour awake per night)<br>2 My sleep was mildly disturbed (1-2 hours awake per night)<br>3 My sleep was moderately disturbed (2-3 hours awake per night)<br>4 My sleep was greatly disturbed (3-5 hours awake per night)<br>5 My sleep was completely disturbed (5-7 hours awake per night)
Social and recreational activities	How have your social and recreational activities been during the past 2 weeks?	0 I have been able to engage in all of my social and recreational activities with no trouble at all<br>1 I have been able to engage in all of my social and recreational activities with difficulty

		2	I have been able to engage in most, but not all, of my social and recreational activities
		3	I have been able to engage in a few of my social and recreational activities
		4	I could hardly engage in any social or recreational activities
		5	I engaged in no social or recreational activities at all
