# Supplementary material for: Development and initial testing of a brief, generic self-reported disability questionnaire: The Universal Disability Index
Source: PLoS One. 2024 May 8;19(5):e0303102. doi: 10.1371/journal.pone.0303102 (PMC11078367; doi:10.1371/journal.pone.0303102)
Supplement: S2 Table — (PDF) [file pone.0303102.s002.pdf]

Table S2. Comparisons of categorical variables between EFA and CFA datasets

Variable	Level	Frequency EFA	Proportion EFA	Percentage EFA	NA Count EFA	Total Count EFA	Frequency CFA	Proportion CFA	Percentage CFA	NA Count CFA	Total Count CFA
sex_birth	Male	58	0.31868132	31.8681319	0	182	51	0.28021978	28.021978	0	182
sex_birth	Female	123	0.67582418	67.5824176	0	182	131	0.71978022	71.978022	0	182
sex_birth	Prefer not to say	1	0.00549451	0.54945055	0	182	NA	NA	NA	0	182
gender_identity	Male	57	0.31318681	31.3186813	0	182	49	0.26923077	26.9230769	0	182
gender_identity	Female	122	0.67032967	67.032967	0	182	131	0.71978022	71.978022	0	182
gender_identity	Non-binary	2	0.01098901	1.0989011	0	182	1	0.00549451	0.54945055	0	182
gender_identity	Prefer not to say	1	0.00549451	0.54945055	0	182	1	0.00549451	0.54945055	0	182
education_age	Aged 16 or less	17	0.09340659	9.34065934	0	182	24	0.13186813	13.1868132	0	182
education_age	Aged 17-19	25	0.13736264	13.7362637	0	182	37	0.2032967	20.3296703	0	182
education_age	Aged 20 or over	109	0.5989011	59.8901099	0	182	98	0.53846154	53.8461538	0	182
education_age	Still in full-time education	30	0.16483516	16.4835165	0	182	23	0.12637363	12.6373626	0	182
education_age	Prefer not to say	1	0.00549451	0.54945055	0	182	NA	NA	NA	0	182
currently_working	Yes	94	0.51648352	51.6483516	0	182	117	0.64285714	64.2857143	0	182
currently_working	No	84	0.46153846	46.1538462	0	182	63	0.34615385	34.6153846	0	182
currently_working	Prefer not to say	4	0.02197802	2.1978022	0	182	2	0.01098901	1.0989011	0	182
job_time	Part-time	31	0.17032967	17.032967	88	182	42	0.23076923	23.0769231	67	182
job_time	Full-time	63	0.34615385	34.6153846	88	182	73	0.4010989	40.1098901	67	182
ethnic_group	White	166	0.91208791	91.2087912	0	182	169	0.92857143	92.8571429	0	182
ethnic_group	Asian or Asian British	2	0.01098901	1.0989011	0	182	2	0.01098901	1.0989011	0	182
ethnic_group	Black or Black British	3	0.01648352	1.64835165	0	182	2	0.01098901	1.0989011	0	182
ethnic_group	Chinese or Chinese British	1	0.00549451	0.54945055	0	182	2	0.01098901	1.0989011	0	182

ethnic_group	Other ethnic group	7	0.03846154	3.84615385	0	182	7	0.03846154	3.84615385	0	182
ethnic_group	Prefer not to say	3	0.01648352	1.64835165	0	182	NA	NA	NA	0	182
other_ethnic_group	Aboriginal	1	0.00549451	0.54945055	175	182	NA	NA	NA	175	182
other_ethnic_group	Amazight	1	0.00549451	0.54945055	175	182	NA	NA	NA	175	182
other_ethnic_group	Bi-racial; half-White, half-Jewish	1	0.00549451	0.54945055	175	182	NA	NA	NA	175	182
other_ethnic_group	Jewish	1	0.00549451	0.54945055	175	182	NA	NA	NA	175	182
other_ethnic_group	mixed Asian and White European	1	0.00549451	0.54945055	175	182	NA	NA	NA	175	182
other_ethnic_group	Mixed white and black Caribbean	1	0.00549451	0.54945055	175	182	NA	NA	NA	175	182
other_ethnic_group	Mixed white asian	1	0.00549451	0.54945055	175	182	NA	NA	NA	175	182
other_ethnic_group	Arab	NA	NA	NA	175	182	1	0.00549451	0.54945055	175	182
other_ethnic_group	British Sikh	NA	NA	NA	175	182	1	0.00549451	0.54945055	175	182
other_ethnic_group	Cornish	NA	NA	NA	175	182	1	0.00549451	0.54945055	175	182
other_ethnic_group	Mixed Asian white	NA	NA	NA	175	182	1	0.00549451	0.54945055	175	182
other_ethnic_group	Mixed Chinese and White	NA	NA	NA	175	182	1	0.00549451	0.54945055	175	182
other_ethnic_group	Mixed white and far east asian	NA	NA	NA	175	182	1	0.00549451	0.54945055	175	182
other_ethnic_group	Mixed White-Arab	NA	NA	NA	175	182	1	0.00549451	0.54945055	175	182
smoker_ever	No	110	0.6043956	60.4395604	0	182	111	0.60989011	60.989011	0	182
smoker_ever	Yes	72	0.3956044	39.5604396	0	182	71	0.39010989	39.010989	0	182
smoker_current	No	52	0.28571429	28.5714286	110	182	51	0.28021978	28.021978	111	182
smoker_current	Yes	20	0.10989011	10.989011	110	182	20	0.10989011	10.989011	111	182
country_residence	Australia	11	0.06043956	6.04395604	0	182	14	0.07692308	7.69230769	0	182
country_residence	Canada	3	0.01648352	1.64835165	0	182	4	0.02197802	2.1978022	0	182
country_residence	Cyprus	4	0.02197802	2.1978022	0	182	6	0.03296703	3.2967033	0	182

country_residence	France	1	0.00549451	0.54945055	0	182	1	0.00549451	0.54945055	0	182
country_residence	Greece	3	0.01648352	1.64835165	0	182	1	0.00549451	0.54945055	0	182
country_residence	Ireland	3	0.01648352	1.64835165	0	182	2	0.01098901	1.0989011	0	182
country_residence	Italy	2	0.01098901	1.0989011	0	182	NA	NA	NA	0	182
country_residence	Switzerland	1	0.00549451	0.54945055	0	182	1	0.00549451	0.54945055	0	182
country_residence	UAE	1	0.00549451	0.54945055	0	182	NA	NA	NA	0	182
country_residence	UK	130	0.71428571	71.4285714	0	182	138	0.75824176	75.8241758	0	182
country_residence	USA	23	0.12637363	12.6373626	0	182	12	0.06593407	6.59340659	0	182
country_residence	NZ	NA	NA	NA	0	182	1	0.00549451	0.54945055	0	182
country_residence	Oman	NA	NA	NA	0	182	1	0.00549451	0.54945055	0	182
country_residence	Portugal	NA	NA	NA	0	182	1	0.00549451	0.54945055	0	182
