# Supplementary material for: Development and initial testing of a brief, generic self-reported disability questionnaire: The Universal Disability Index
Source: PLoS One. 2024 May 8;19(5):e0303102. doi: 10.1371/journal.pone.0303102 (PMC11078367; doi:10.1371/journal.pone.0303102)
Supplement: S3 Table — (PDF) [file pone.0303102.s003.pdf]

S3 Table. Test statistics for comparisons of categorical variables between EFA and CFA datasets

Variable	Test	X-square	P_Value	DF
sex_birth	Chi-Square	2.68371502	0.26135974	2
gender_identity	Chi-Square	3.64342961	0.93327714	9
education_age	Chi-Square	17.2772238	0.13946329	12
currently_working	Chi-Square	0.911238	0.92294304	4
job_time	Chi-Square	0.03671787	0.84804059	1
ethnic_group	Chi-Square	9.7734013	0.87819079	16
smoker_ever	Chi-Square	0.02214375	0.88170534	1
smoker_current	Chi-Square	6.05E-32	1	1
