# Supplementary material for: Development and initial testing of a brief, generic self-reported disability questionnaire: The Universal Disability Index
Source: PLoS One. 2024 May 8;19(5):e0303102. doi: 10.1371/journal.pone.0303102 (PMC11078367; doi:10.1371/journal.pone.0303102)
Supplement: S4 Table — (PDF) [file pone.0303102.s004.pdf]

**S4 Table. Comparisons of continuous variables between EFA and CFA datasets**

| Variable | Mean EFA | Median EFA | SD EFA | CI Low EFA | CI High EFA | NA Count EFA | Mean CFA | Median CFA | SD CFA | CI Low CFA | CI High CFA | NA Count CFA |
|---|---|---|---|---|---|---|---|---|---|---|---|---|
| age | 43.35 | 44 | 16.67 | 21 | 75.48 | 0 | 46.30 | 47.5 | 17.67 | 21 | 77 | 0 |
