# Supplementary material for: Development and initial testing of a brief, generic self-reported disability questionnaire: The Universal Disability Index
Source: PLoS One. 2024 May 8;19(5):e0303102. doi: 10.1371/journal.pone.0303102 (PMC11078367; doi:10.1371/journal.pone.0303102)
Supplement: S5 Table — (PDF) [file pone.0303102.s005.pdf]

S5 Table. Test statistics for comparisons of continuous variables between EFA and CFA subsets

Variable	Test	W	P-value
age	Wilcoxon	15903	0.51160388
