# Supplementary material for: Development and initial testing of a brief, generic self-reported disability questionnaire: The Universal Disability Index
Source: PLoS One. 2024 May 8;19(5):e0303102. doi: 10.1371/journal.pone.0303102 (PMC11078367; doi:10.1371/journal.pone.0303102)
Supplement: S6 Table — (PDF) [file pone.0303102.s006.pdf]

S6 Table. Correlation matrix of disability measures for subset of participants with non-zero current pain intensity

	UDI 8 total	UDI 7 total	UDI 6 total	GARS total	GCPS disability
**UDI 7 total**	0.992 *** (0.990, 0.994)				
**UDI 6 total**	0.986*** (0.982, 0.989)	0.994 *** (0.993, 0.995)			
**GARS total**	0.812 *** (0.767, 0.850)	0.828 *** (0.788, 0.863)	0.820 *** (0.779, 0.857)		
**GCPS disability**	0.713 *** (0.640, 0.778)	0.706 *** (0.631, 0.773)	0.705 *** (0.628, 0.773)	0.614 *** (0.528, 0.693)	
**GCPS pain intensity**	0.546 *** (0.458, 0.628)	0.536 *** (0.443, 0.619)	0.537 *** (0.448, 0.622)	0.460 *** (0.361, 0.559)	0.823 *** (0.781, 0.860)
