# Supplementary figures and images for: Development and initial testing of a brief, generic self-reported disability questionnaire: The Universal Disability Index
Source: PLoS One. 2024 May 8;19(5):e0303102. doi: 10.1371/journal.pone.0303102 (PMC11078367; doi:10.1371/journal.pone.0303102)

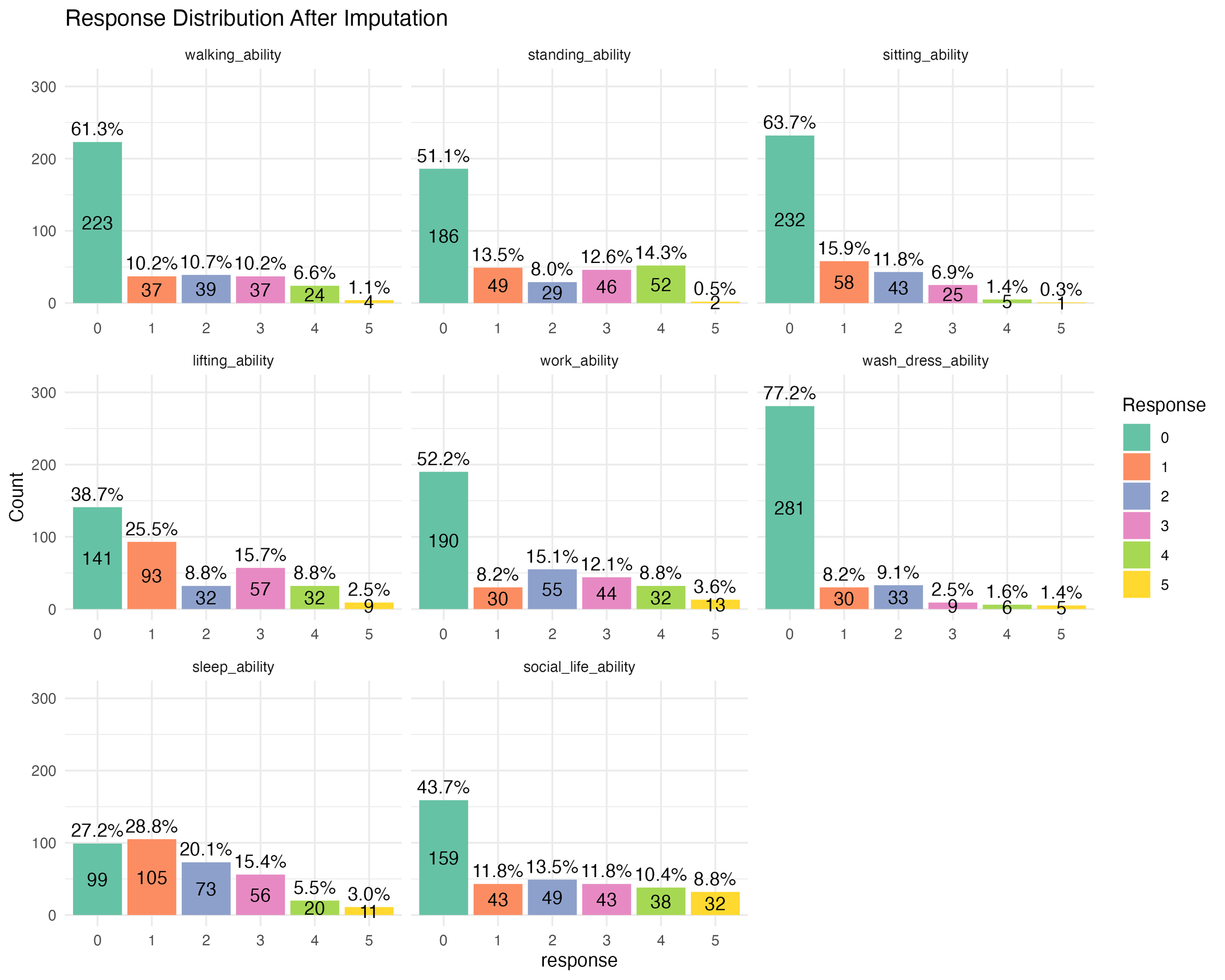

Supplement: S1 Fig — (TIF) [file pone.0303102.s007.tif]

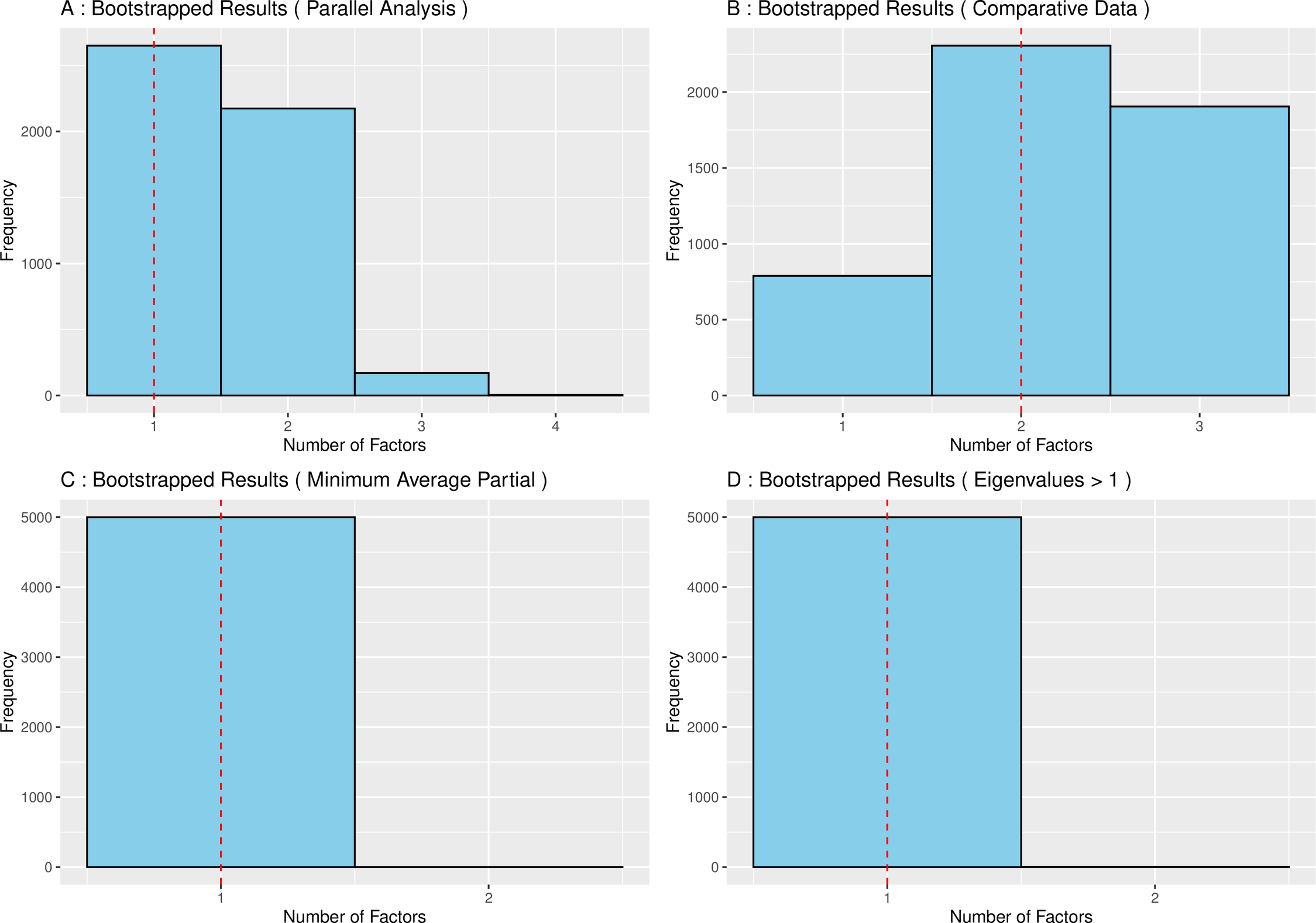

Supplement: S2 Fig — (TIF) [file pone.0303102.s008.tif]

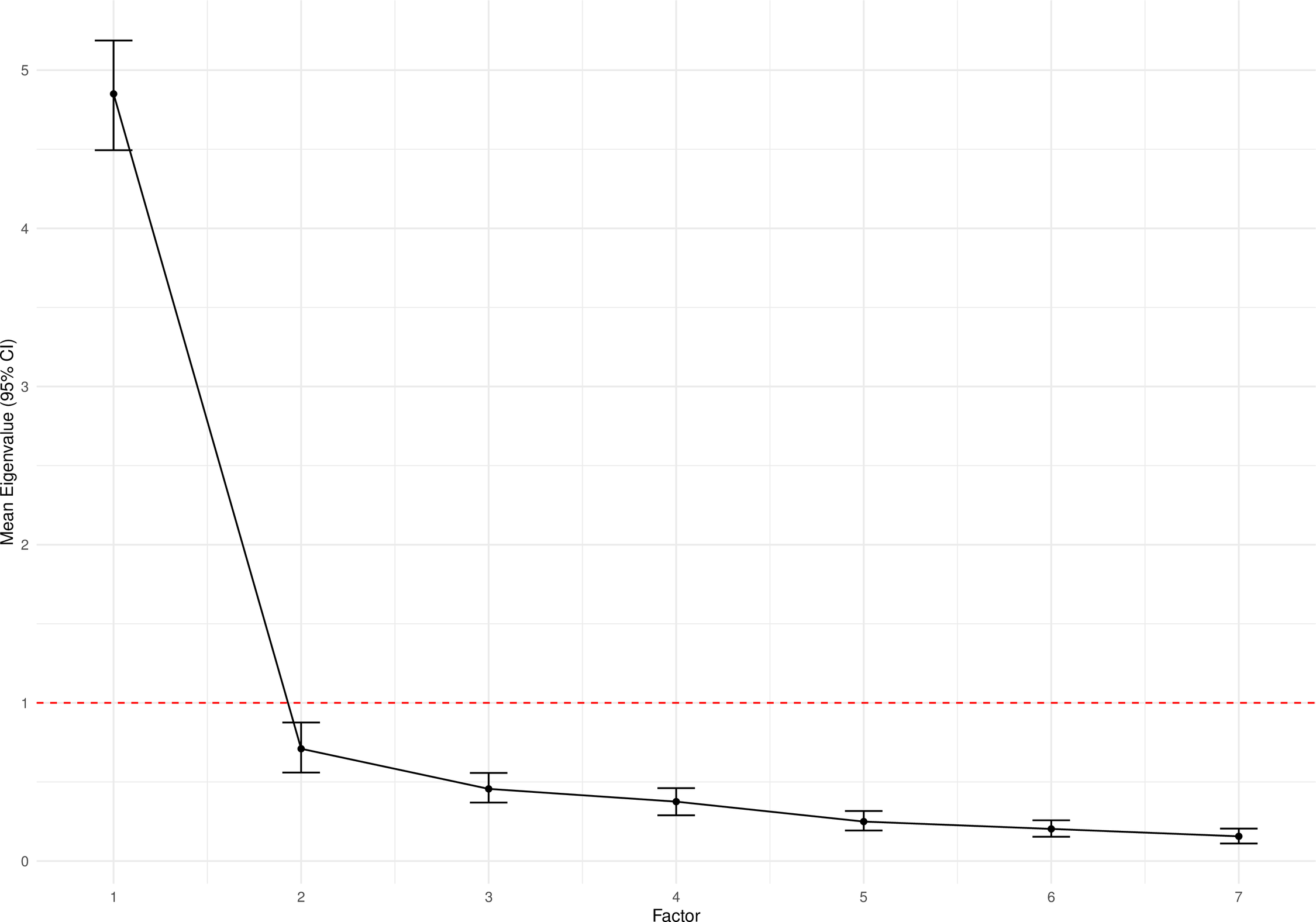

Supplement: S3 Fig — (TIF) [file pone.0303102.s009.tif]

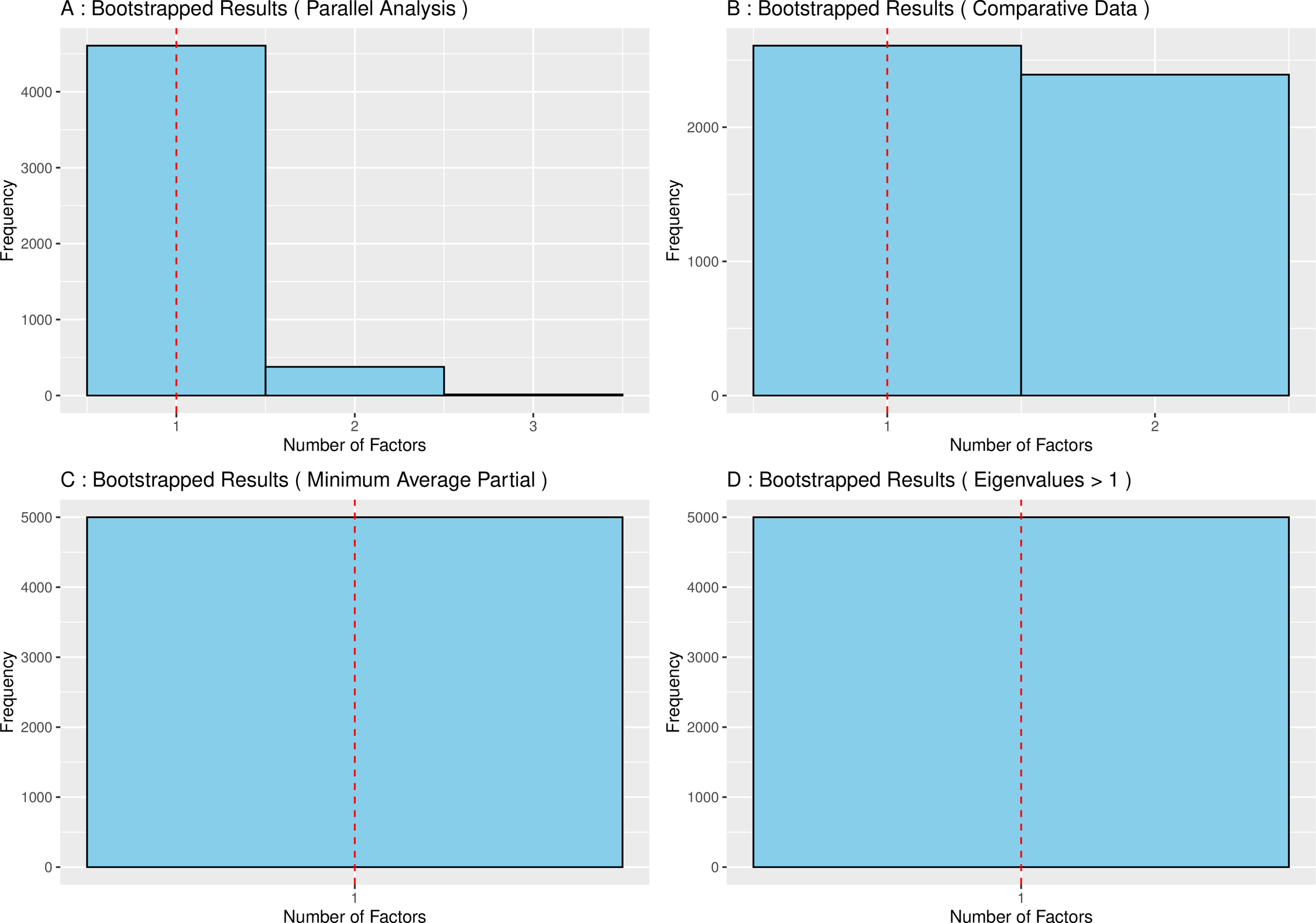

Supplement: S4 Fig — (TIF) [file pone.0303102.s010.tif]

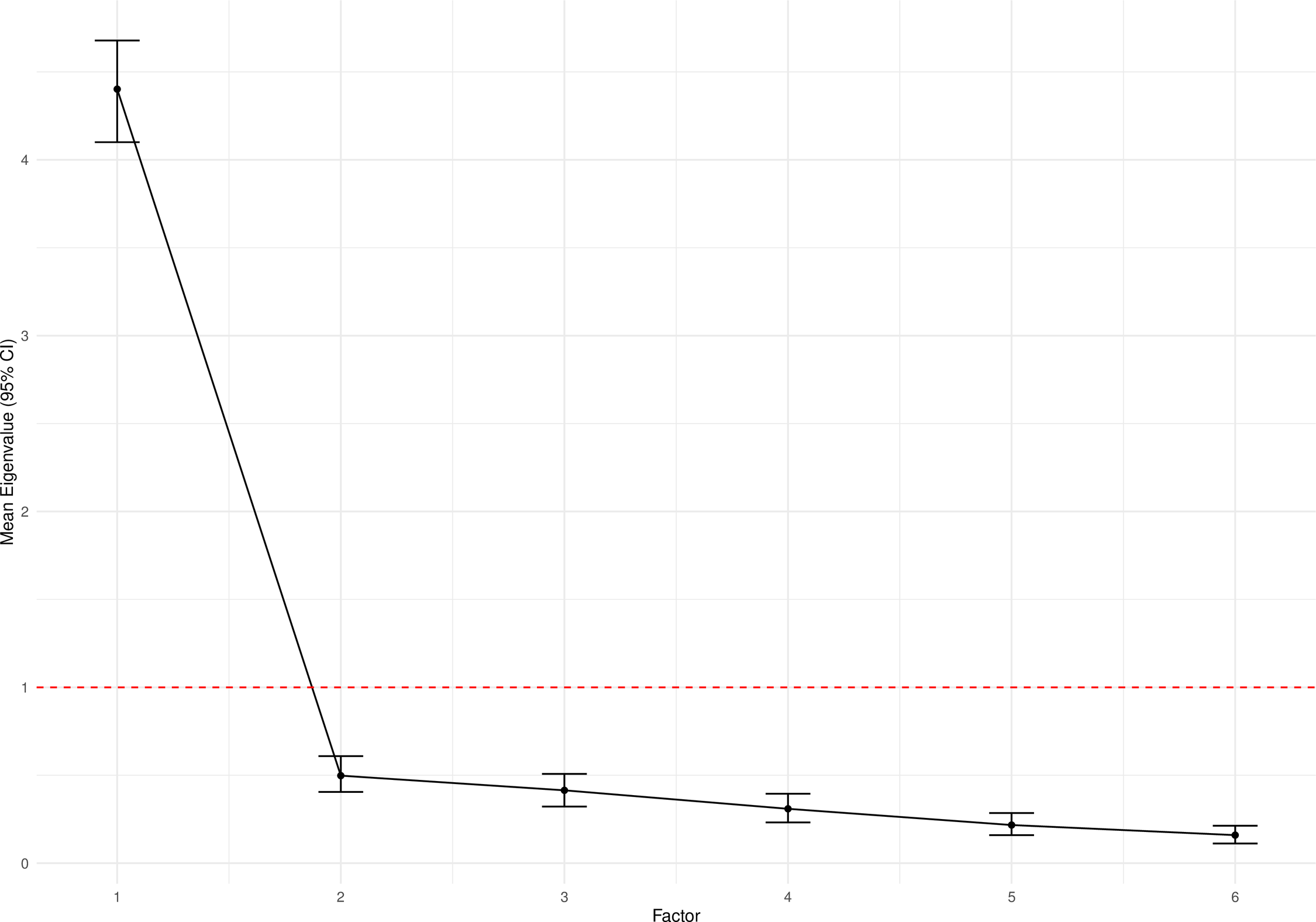

Supplement: S5 Fig — (TIF) [file pone.0303102.s011.tif]
