# Supplementary material for: Development and initial testing of a brief, generic self-reported disability questionnaire: The Universal Disability Index
Source: PLoS One. 2024 May 8;19(5):e0303102. doi: 10.1371/journal.pone.0303102 (PMC11078367; doi:10.1371/journal.pone.0303102)
Supplement: Attachment — Submitted filename: Response to reviewers.docx [file pone.0303102.s012.docx]

**Response to reviewers**

Reviewer #1: Congratulations to the authors for a very well conducted and adequately reported study; however, there are some scopes of modification and improving the manuscript.

1. Introduction is divided into too many paragraphs. Kindly merge those meaningfully into lesser number of paragraphs.

Thank you for this suggestion. Having tried to merge paragraphs of the introduction in different ways, it has difficult to make this work. The first two paragraphs were merged, but upon re-reading them, this merger does not work well. Based on the following guidance, we feel the original paragraph divisions probably should be retained.

When to start a new paragraph (<https://libguides.newcastle.edu.au/writing-paragraphs/structure>):

- Start new main points or new ideas in a new paragraph. If you have an extended idea across multiple paragraphs, each new point within that idea should have its own paragraph.
- Use a new paragraph to introduce a contrasting or different position. Use a clear topic sentence to identify the main idea.
- If the paragraph becomes too long or the material is overly complex, you will need to create a break to make your writing more readable. Try splitting long paragraphs into two shorter paragraphs. This means you will need to write a new topic sentence at the start of the new paragraph.
- Introductions and conclusions are usually written as separate paragraphs.

2. How was the sample size determined? Please elaborate.

Factor analysis was the primary analysis of the study that determined sample size requirements. Sample sizes for factor analysis are unfortunately not straightforward but, in general, greater numbers do provide increased accuracy of estimates, which is why we used a minimum target sample size and did not specify an upper limit. The minimum target sample size in this study to perform both exploratory factor analysis (EFA) and confirmatory factor analysis (CFA) (320 respondents) was based on stated requirements for each stage (minimum of 20 responses on each of the 8 UDI items). This was explicitly mentioned on page 12 of the original manuscript:
 *Depending on the size of the available sample, at least 20 cases per UDI item [1] (i.e., at least 160 cases) were to be selected for a training dataset for exploratory factor analysis (EFA). The number was also anticipated to be necessary for a validation dataset to perform confirmatory factor analysis (CFA) [2]. If the study sample was sufficiently large (i.e., 320 or more useable responses), the sample would be randomly divided into training and validation subsets; otherwise, the full dataset would be used only for EFA, and CFA would not be performed. In the event of data sub-setting, the distribution of participant characteristics between the two subsets would be compared.*

And in results, it was stated (page 20):

*With 364 participants providing UDI data, there were sufficient participants to subset half (182 cases) as a training dataset (providing 22.75 values per UDI item for EFA) and the same number of cases for validation (CFA).*

3. Kindly provide the diagram of the CFA model.

Thank you for suggesting this. The path diagrams for the 8-item, 7-item and 6-item CFA models have now been added as an additional figure (Fig 4. a, b, c).

4. Please rephrase the 1st sentence of conclusion.

Thank you for pointing out this opportunity to improve the manuscript. The original first sentence of the conclusion was:

“*Through an evaluation using rigorous methodology, a brief, generic self-reported disability questionnaire was found to be valid and possess good psychometric properties.”*

This has now been rephrased to:

“*The Universal Disability Index (UDI) is a brief, generic self-reported disability questionnaire that appears to be valid and to possess good psychometric properties*.”

Many thanks indeed to Reviewer 1 for spending their valuable time in providing feedback on this work.

**Reviewer #2:**

This was a very well-written manuscript and the statistical analysis for validation of the UDI was reported meticulously. The authors have provided reasonable justifications for development of a universal tool to measure disability across different conditions. There are some queries and suggestions that I hope the authors can address.

Many thanks for these positive comments.

1. Bearing in mind that there are various types of disability, which may include physical, mental, emotional and social disability. How well can the UDI be relevant to different types of disability such as those due to mental or emotional illnesses? It may be necessary to revise that UDI is aimed at measuring universal physical disability rather than all types of disability.

Thank you for this thoughtful comment. This issue was considered in depth so Reviewer 2’s comment is appreciated.

One of the major reasons for deliberately including broad ADL categories such as ‘work and daily routine', ‘social and recreational activities’ and ‘sleeping’ in the UDI was to create a measure of disability caused by physical impairments and/or mental health problems, including emotional and behavioural factors. Note that the question phrasing of the UDI did not follow that of the ODI or NDI, instead taking the form of: “*How much have you been able to* …”, and therefore being deliberately wide-ranging and much more inclusive than these other questionnaires. Hence, an entirely unattributed, generic measure such as the UDI should be able to capture disability manifesting from non-physical causes. Indeed, there are several examples where mental health can manifest as interference with activities of daily living. For example, when measured using disability-adjusted life-years (DALYS) (the sum of years lived with disability and years of life lost), mental health disorders have remained among the top ten leading causes of burden worldwide for the past 30 years [3].

In this sense, the UDI reflects the approach of the World Health Organization (embodied within the ICF) as being neutral to aetiology, instead placing the emphasis on function rather than a specific condition or disease. In the ICF, disability refers to functional or structural impairments, limitations in personal activities, and restrictions on social participation within the context of environmental factors. In other words, disability denotes the negative outcomes of the interaction between an individual's health condition and contextual factors (environmental and personal factors) that may affect their health.

In the current study, diversity of health condition amongst participants was deliberately sought by targeting online support groups with on social media platforms, including those for pain problems, mental health conditions, and individuals experiencing chronic fatigue, as well as including healthy people in the general population. Therefore, the derived factor structures and associated loading estimates should be robust for a wide variety of conditions (see comparison of the full dataset with those with non-zero pain below). Obviously, future studies in groups with specific health conditions are now needed.

1. The UDI items were derived from the Oswestry Disability Index and Neck Disability Index which were validated to measure disability due to pain. The item for pain was not included into the UDI. Was there a reason for this?

The item for pain was not included amongst the UDI items because of the desire to create a generic, condition-generic disability questionnaire based on ADLs.

2. There are other established generic disability tools such as Modified Barthel Index , or Katz Index of Independence in Activities of Daily Living. These are used in various types of conditions as well. How does the UDI compare with these tools? Thus far, the authors have discussed the comparison of UDI with GARS and very few other tools.

The motivation for creating the UDI was that there are surprisingly few generic self-reported disability questionnaires available. As mentioned in the existing introduction (pages 5-6):

“*Surprisingly, relatively few generic self-reported disability questionnaires are currently available. Of those that are, there is either a focus on a specific aspect of disability, such as task independence [4], they incorporate complex ‘instrumental’ activities that may not be universally relevant [4-6], or they are very lengthy [5].*”

Another reason for not adding more generic questionnaires to the survey (for example, the SF-36v2 and EQ-5D-5L were considered) was to not burden participants with too many questions and too lengthy a survey. Hence, the generic GARS and the pain-specific GCPS were deemed to be appropriate comparators in this initial evaluation.

To address the specific disability measures suggested here by Reviewer 2, the original Barthel Index [7] assesses ten common ADLs, including basic mobility (original version items: feeding, bathing, grooming, dressing, bowel, bladder, toilet use, transfers from bed-to-chair-and-back, mobility on level surfaces, stair negotiation). Neither original [7] nor modified versions [8] of the Barthel Index are self-reported questionnaires and are instead scored through observation by another person who is familiar with the ADLs being assessed. Similarly, the Katz Index of Independence in Activities of Daily Living is another tool that is scored by a third-party observer (items: bathing, dressing, toileting, transferring, continence, and feeding). This makes these in-person observation instruments impossible to test in a self-reported online survey, such as that used in the current study, but they are potential candidates for future validation studies that utilise a different methodology (i.e. not just self-reported questionnaires).

In addition, the ADLs incorporated in the Barthel Index and those of the Katz Index are typically only affected at the more severe end of the disability spectrum (e.g. following a stroke or other neurological disorder). Whilst some of these ADLs could feasibly be affected by painful conditions and health issues that give rise to fatigue, they are less likely to be markedly affected by mental health issues such as depression or anxiety (unless at the most extreme levels). By contrast, the UDI deliberately includes broader categories, such as ‘work and daily routine’, ‘social and recreational activities’ and ‘sleeping’, which are known to be affected by such conditions (even at mild or moderate severities) yet still has the capability to measure more severe disability.

3. The UDI was compared against GARS and GCPS. Comparison with GARS is suitable. However, I would like to know why the authors chose a pain-related scale for comparison. While pain is prevalent, not all disabilities are associated with pain. Patients with neurological disorders with loss of sensation may not experience pain but they still have disability.

To a large extent, the range and choice of comparator disability scales was limited by their availability. By targeting independence of ADL performance, the GARS is condition-generic, even though a measure of independence is likely to be most relevant to the more severe end of the disability spectrum. By contrast, pain is very common in the general population [9], so it was absolutely essential to test the ability of the UDI to measure pain0related disability. Hence, the UDI was compared to a well-established pain-specific measure of disability. Given the decision to recruit a sample from the general population, there were very few pain-related disability questionnaires to choose from that were not anatomically restricted (e.g. not restricted to the lower back, neck, or shoulder, for example) and the Graded Chronic Pain Scale possessed several other virtues (it was brief, has good psychometric properties, and is the most widely used of those that met the other criteria). In future studies, the UDI will need to be compared to various condition/disease-specific instruments in relevant populations to further test the breadth/limits of its validity.

Therefore, did the authors select out participants who had pain for the analysis which compared the UDI and GCPS? Otherwise, those without pain might have discrepantly lower scores for GCPS. This could have affected the correlation between UDI and GCPS pain intensity.

Many thanks for this suggestion. Pearson correlations were recalculated for the subset of participants with a non-zero current pain intensity score that had completed at least one item of the UDI (n=276). In this sensitivity analysis, correlations between disability measures were very similar to those of the full dataset (see tables below) and the correlation matrix for this subset (S6 Table) has been added to the Supporting Information.

**Table 5. Correlation matrix of disability measures**

|  | **UDI 8 total** | **UDI 7 total** | **UDI 6 total** | **GARS total** | **GCPS disability** |
| --- | --- | --- | --- | --- | --- |
| **UDI 7 total** | 0.993 ***  (0.991, 0.994) |  |  |  |  |
| **UDI 6 total** | 0.987 ***  (0.985, 0.990) | 0.995 ***  (0.994, 0.996) |  |  |  |
| **GARS total** | 0.827 ***  (0.791, 0.859) | 0.840 ***  (0.807, 0.869) | 0.834 ***  (0.800, 0.864) |  |  |
| **GCPS disability** | 0.735 ***  (0.673, 0.791) | 0.722 ***  (0.655, 0.782) | 0.719 ***  (0.651, 0.779) | 0.631 ***  (0.556, 0.702) |  |
| **GCPS pain intensity** | 0.611 ***  (0.543, 0.677) | 0.593 ***  (0.519, 0.659) | 0.586 ***  (0.510, 0.652) | 0.500 ***  (0.419, 0.580) | 0.844 ***  (0.808, 0.876) |

* *p* < 0.05

** *p* < 0.01

*** *p* < 0.001

**S6 Table. Correlation matrix of disability measures for subset of participants with non-zero current pain intensity**

|  | **UDI 8 total** | **UDI 7 total** | **UDI 6 total** | **GARS total** | **GCPS disability** |
| --- | --- | --- | --- | --- | --- |
| **UDI 7 total** | 0.992 ***  (0.990, 0.994) |  |  |  |  |
| **UDI 6 total** | 0.986***  (0.982, 0.989) | 0.994 ***  (0.993, 0.995) |  |  |  |
| **GARS total** | 0.812 ***  (0.767, 0.850) | 0.828 ***  (0.788, 0.863) | 0.820 ***  (0.779, 0.857) |  |  |
| **GCPS disability** | 0.713 ***  (0.640, 0.778) | 0.706 ***  (0.631, 0.773) | 0.705 ***  (0.628, 0.773) | 0.614 ***  (0.528, 0.693) |  |
| **GCPS pain intensity** | 0.546 ***  (0.458, 0.628) | 0.536 ***  (0.443, 0.619) | 0.537 ***  (0.448, 0.622) | 0.460 ***  (0.361, 0.559) | 0.823 ***  (0.781, 0.860) |

* *p* < 0.05

** *p* < 0.01

*** *p* < 0.001

A line of text was added to the methods of the main text to describe this sensitivity analysis (page 16). A line of text was added to the methods of the main text to describe the results of this sensitivity analysis (page 16):

“*In addition, as a sensitivity analysis, correlations were also assessed in the subset of participants with a non-zero ‘current’ pain intensity score (one of the GCPS items)*.”

In the results (page 25):

“*In the subset of participants with non-zero current pain and at least one completed UDI item (n=276), point estimates (means) of these correlations were very similar to those of the full dataset (full results: the UDI8 was strongly correlated with both the GARS (r = 0.812 [0.767, 0.850]) and the GCPS disability (r = 0.713 [0.640, 0.778]). Full details of this sensitivity analysis are provided in supporting information.*”

And in the discussion (page 30):

“*The similarity in magnitudes of correlation coefficients for the full dataset with the subset of participants with non-zero current pain supports the validity of the UDI in capturing important aspects of disability, irrespective of the potential origins of this disability. It also indicates that the statistical models from which estimates were gained in this study are likely to be stable.*”

Besides presenting the mean scores for their pain intensity in Table 1, do present the categorical results as well. e.g. How many n(%) had no pain, mild pain, moderate pain etc.

There is no agreed categorisation for descriptors of mild, moderate, severe pain intensity, beyond the preference of individual authors. This is partly because divisions in numerical pain rating scales are difficult to interpret [10]. In addition, the ‘characteristic pain intensity’ score of the GCPS (‘GCPS pain intensity), utilised here, is calculated as the mean of three different 0-10 numerical pain rating scales (current, worst, and perceived average), which makes it difficult to categorise meaningfully. This is why the standard deviation was used as an appropriate measure of pain intensity dispersion in Table 1. To offer categories with some validity, ‘Currently pain-free’ and ‘Currently experiencing pain’ (non-zero on the single ‘current’ pain intensity’ item of the GCPS) have been added to Table 1.

| Current pain, n (%)** | Experiencing pain, 281 (69.9)  Pain-free, 90 (22.4)  Missing, 31 (7.7) |
| --- | --- |

4. Table 1, I noticed that educational attainment was presented as age of having left formal education. This is probably personal preference but why didn't the authors present it according to levels of formal education rather than on age of leaving full time education? This is however a minor comment.

Thank you for this question. As you mention, age of leaving full-time education is one of several ways to evaluate educational attainment. We did not want to appear intimidating or judgmental by directly asking respondents what qualifications they had (high school, undergraduate degree, higher degree, etc), so opted for this less direct approach.

5. Some results which have been presented in the tables do not need to be repeated in the main text, as the manuscript is already very long and wordy.

Repetition has been avoided wherever possible, with the text focusing only on the main points. Efforts have been made to further improve this. Please specify if you feel that any remaining text is redundant and should be removed/edited.

6. Table 7 displays numbers with 9 decimal places. Perhaps this can be converted into max 3 decimal points.

Thank you for this recommendation. Table 7 has now been revised to display values rounded to 4 decimal points. The values were originally presented in their raw form because 95% confidence intervals were so narrow that they would frequently be identical to the mean score if rounded to 3 decimal places.

I hope these comments are helpful for the authors to improve on the manuscript. Good job!

Many thanks indeed to Reviewer 2 for spending their valuable time in providing feedback on this work.

**References**

1.	Costello AB, Osborne JW. Best practices in exploratory factor analysis: Four recommendations for getting the most from your analysis. Practical Assessment, Research, & Evaluation. 2005;10:1-9.

2.	Wolf EJ, Harrington KM, Clark SL, Miller MW. Sample Size Requirements for Structural Equation Models: An Evaluation of Power, Bias, and Solution Propriety. Educ Psychol Meas. 2013;76(6):913-34. doi: 10.1177/0013164413495237. PubMed PMID: 25705052; PubMed Central PMCID: PMCPMC4334479.

3.	Collaborators GBDMD. Global, regional, and national burden of 12 mental disorders in 204 countries and territories, 1990-2019: a systematic analysis for the Global Burden of Disease Study 2019. Lancet Psychiatry. 2022;9(2):137-50. Epub 20220110. doi: 10.1016/S2215-0366(21)00395-3. PubMed PMID: 35026139; PubMed Central PMCID: PMCPMC8776563.

4.	Suurmeijer TP, Doeglas DM, Moum T, Briancon S, Krol B, Sanderman R, et al. The Groningen Activity Restriction Scale for measuring disability: its utility in international comparisons. Am J Public Health. 1994;84(8):1270-3. doi: 10.2105/ajph.84.8.1270. PubMed PMID: 8059884; PubMed Central PMCID: PMCPMC1615477.

5.	Johnson N, Barion A, Rademaker A, Rehkemper G, Weintraub S. The Activities of Daily Living Questionnaire: a validation study in patients with dementia. Alzheimer Dis Assoc Disord. 2004;18(4):223-30. PubMed PMID: 15592135.

6.	Pfeffer RI, Kurosaki TT, Harrah CH, Jr., Chance JM, Filos S. Measurement of functional activities in older adults in the community. J Gerontol. 1982;37(3):323-9. doi: 10.1093/geronj/37.3.323. PubMed PMID: 7069156.

7.	Mahoney FI, Barthel DW. Functional Evaluation: The Barthel Index. Md State Med J. 1965;14:61-5. PubMed PMID: 14258950.

8.	Shah S, Vanclay F, Cooper B. Improving the sensitivity of the Barthel Index for stroke rehabilitation. J Clin Epidemiol. 1989;42(8):703-9. doi: 10.1016/0895-4356(89)90065-6. PubMed PMID: 2760661.

9.	Fayaz A, Croft P, Langford RM, Donaldson LJ, Jones GT. Prevalence of chronic pain in the UK: a systematic review and meta-analysis of population studies. BMJ Open. 2016;6(6):e010364. Epub 2016/06/22. doi: 10.1136/bmjopen-2015-010364. PubMed PMID: 27324708; PubMed Central PMCID: PMCPMC4932255.

10.	Walton DM, Elliott JM, Salim S, Al-Nasri I. A reconceptualization of the pain numeric rating scale: Anchors and clinically important differences. J Hand Ther. 2018;31(2):179-83. Epub 2018/02/13. doi: 10.1016/j.jht.2017.12.008. PubMed PMID: 29433765.
