# Supplementary material for: Development and initial testing of a brief, generic self-reported disability questionnaire: The Universal Disability Index
Source: PLoS One. 2024 May 8;19(5):e0303102. doi: 10.1371/journal.pone.0303102 (PMC11078367; doi:10.1371/journal.pone.0303102)
Supplement: Attachment — Submitted filename: Response to reviewers_submission3.docx [file pone.0303102.s013.docx]

**Response to reviewers**

**Reviewer #1:**

No comments provided. Thanks you for your original review.

**Reviewer #2:**

Thank you for addressing my comments in your response letter. The sensitivity analysis for pain has been incorporated into the manuscript. However, some of the other comments were answered in the response to reviewer but not incorporated into the manuscript. I apologise if this was not made clear in the previous review.
I accept the well-written rebuttals. I would suggest that some of the explanations should be included into the manuscript itself.

1. Include : Although pain was an item in the original Oswestry Disability Index and Neck Disability Index, the item for pain was not included in the UDI to ensure that the UDI was applicable to generic conditions.

Thank you for clarifying. The following text has been added to the manuscript (p10) to explain why items were selected and others not:

*Although pain is an item in both the ODI and NDI, a ‘pain’ item was not incorporated into the UDI because of the desire to create a condition-generic questionnaire based on ADLs. Instead, items related to everyday activities (e.g., walking, standing, lifting) or life situations (e.g., social, recreational, or work activities) were chosen so that the UDI aligned with the WHO’s ICF framework [5]. Similarly, …*

2. Include a brief explanation that the UDI was aimed for patient self-report and hence different from measures such as the Barthel Index or Katz Index of Independence which were assessed by a third-party observer. It would also mean that the UDI can only be suitable for respondents who have adequate cognitive capacity to respond, as MBI and Katz Index can be used for patients with cognitive impairment.

Thank you for highlighting this important point. The introduction did state:

*If the assessee has very severe levels of disability, in-person assessment may be the only option. However, people with all but the most severe levels of disability are often able to self-report their capabilities and restrictions through questionnaires.*

Nevertheless, the following text has been added to the manuscript to make this important point more explicit (p33):

*As with any self-reported questionnaire, the UDI requires that the respondent possess sufficient mental capacity to understand and respond to the questions. If this criterion is unmet, disability can only be assessed using third party observation, with instruments such as the Katz Index of Independence in Activities of Daily Living [6] or the Barthel Index [7]. On the other hand, third-party observations of ADLs are difficult to perform remotely, and currently require an observer who is trained to be familiar with the ADLs being assessed.*

3. Can the image quality for Figure 1 be improved?

The original resolution of Figure 1 should have been adequate, even as a raster image (see below). The Editorial Manager website PDF rendering tends to reduce this in the review documents. Please be reassured that the resolution of submitted images will be adequate for publication.

Thank you.

Thank you for taking time to review the manuscript again.

**Reviewer #3:** 

Thank you for providing a chance to review this manuscript.

Keywords: Keywords are generally 4-6, the current summary is not appropriate, please reconsider.

The keywords have been reduced to the following:

Disability; Questionnaire; Patient-Reported Outcome Measures (PROMS); Activities of Daily Living (ADLs); Factor analysis

I am sorry to say that I have to reject the author's manuscript. On the one hand, it is puzzling that I did not receive any review comments from the authors in response, as well as no corrections in the manuscript for the problems I presented.

I can only apologise for this. However, your comments from the first review were not included in the information provided to me by the journal. Upon becoming aware (only in this second review) that a third reviewer had provided feedback, I immediately asked the journal office (by email) to send these comments to me (which were not evident within the EM website) and to let you know what had occurred. Unfortunately, despite waiting more than a month for a reply, I have not received any email response so must assume that this request has not been carried out by the journal. Obviously, I am happy to attend to these comments if you are happy to provide these again.

On the other hand, RMSEA < 0.08 represents a fair fit. But in your manuscript, RMSEA > 0.1 indicates that there are problems with the model structure and that the model does not explain the observed data well.

Thank you for this comment. As you will know, it is important to consider the collective evidence from multiple fit indices when performing CFA. The RMSEA values, while important to note (as we have in the results, limitations and the abstract), must be interpreted within the context of the entire model fit assessment and the theoretical framework guiding our study [1-3]. While mean RMSEA values from the three bootstrapped CFA models (0.12, 0.14, and 0.12) exceeded the conventional 0.08 threshold, all other fit indices – Chi square, CFI, TLI, SRMR, AIC, and BIC – all met their respective thresholds and thereby consistently indicated a good model fit. This broad agreement suggests robust model validity. Additionally, the lower bounds of our RMSEA confidence intervals approached and even crossed the 0.08 threshold (0.12 [0.08, 0.17], 0.14 [0.09, 0.19], 0.12 [0.06, 0.19]), suggesting an acceptable fit within statistical variability. RMSEA is known to be sensitive to various factors, including model specification and sample characteristics. Unfortunately, the nature of this variabliity can be complex and is not necessarily linear. Our study, with a moderate sample size of 334, could have resulted in variable RMSEA values. In addition, the deliberate sampling of heterogenous levels of disability might also have influenced RMSEA values. As mentioned in the ‘limitations’ section of the manuscript, testing of the UDI in more homogenous clinical samples is now necessary. Importantly, given the solid theoretical grounding of the UDI, based as it is on theoretically sound, well-tested and well-established existing disability questionnaires (the ODI and NDI), publication of appropriately investigated results that might be regarded as ‘negative’ by some is crucial for science [4, 5]. Hence, I have to strongly oppose the argument that the value of this single metric is a grounds for not publishing this study; a position that PLOS explicitly supports: <https://everyone.plos.org/2020/04/06/filling-in-the-scientific-record-the-importance-of-negative-and-null-results/>.

Thank you and my best,
Your reviewer

Thank you for taking time to review the manuscript.

**References**

1.	Lai K, Green SB. The Problem with Having Two Watches: Assessment of Fit When RMSEA and CFI Disagree. Multivariate Behavioral Research. 2016;51(2-3):220-39. doi: 10.1080/00273171.2015.1134306.

2.	Rigdon EE. CFI versus RMSEA: A comparison of two fit indexes for structural equation modeling. Structural Equation Modeling: A Multidisciplinary Journal. 1996;3(4):369-79. doi: 10.1080/10705519609540052.

3.	Graham JM, Guthrie AC, Thompson B. Consequences of Not Interpreting Structure Coefficients in Published CFA Research: A Reminder. Structural Equation Modeling: A Multidisciplinary Journal. 2003;10(1):142-53. doi: 10.1207/S15328007SEM1001_7.

4.	Ioannidis JP. Why most published research findings are false. PLoS Med. 2005;2(8):e124. Epub 20050830. doi: 10.1371/journal.pmed.0020124. PubMed PMID: 16060722; PubMed Central PMCID: PMCPMC1182327.

5.	Young NS, Ioannidis JP, Al-Ubaydli O. Why current publication practices may distort science. PLoS Med. 2008;5(10):e201. doi: 10.1371/journal.pmed.0050201. PubMed PMID: 18844432; PubMed Central PMCID: PMCPMC2561077.
