# Supplementary material for: Development and initial testing of a brief, generic self-reported disability questionnaire: The Universal Disability Index
Source: PLoS One. 2024 May 8;19(5):e0303102. doi: 10.1371/journal.pone.0303102 (PMC11078367; doi:10.1371/journal.pone.0303102)
Supplement: Attachment — Submitted filename: Response to reviewers_submission3.docx [file pone.0303102.s014.docx]

**Reviewer comments**

***Reviewer #2:***

Thank you for addressing all the points in the review. Congratulations. I look forward to see the published version soon.

Many thanks for your time and effort in providing constructive comments and suggestions.

***Reviewer #3:***

ID: PONE-D-23-31316R2

Title: Development and initial testing of a brief, generic self-reported disability questionnaire: the Universal Disability Index

Thank you for providing a chance to review this manuscript.

Detailed information:

Abstract

Background: Compared with the previous universal disability scale, what are the innovations of the scale in this paper?

I view the UDI as novel (and needed) because there are very few, if any, existing generic disability questionnaires. The primary comparator questionnaire used in this study was the Groningen Activity Restriction Scale (GARS), but this measures *independence* during ADLs, which is similar to – but not the same as – self-reported *ability* in ADL performance. Many, if not most, disease-specific disability questionnaires use self-reported *ability* in ADL performance. Hence, the UDI is an attempt to fill this gap and was created using the wording of well-established disease-specific questionnaires that use concrete examples of ‘normal’ ADL performance (i.e. the Oswestry Disability Index [ODI] and the Neck Disability Index [NDI]).

The above was stated in the introduction as:

“Surprisingly, relatively few generic self-reported disability questionnaires are currently available. Of those that are, there is either a focus on a specific aspect of disability, such as task independence [19], they incorporate complex ‘instrumental’ activities that may not be universally relevant or might be outdated [11, 19, 34], or they are very lengthy [11].”

In the abstract, this was briefly summarised as: “Few generic self-reported disability questionnaires exist. Yet, these promise a broader and more comparable measure of disability than disease-specific instruments.”

Following the suggestion of Reviewer #3, I have attempted to increase the precision of this abstract wording to “A brief, generic self-reported disability questionnaire that promises a broader and more comparable measure of disability than disease-specific instruments does not currently exist.”

I have also added the following sentence (lines 226-228) to provide additional justification for using concrete examples within the UDI response options (as per the ODI and NDI):

“Concrete examples of ADL ability were chosen to ensure consistency between levels of reported (dis)ability, rather than asking respondents to gauge their recent ability compared to their historical levels, which would inevitably vary between individuals.”

Conclusions: The conclusion is not rigorous.

The current conclusion in the abstract is as follows:

“A brief, generic self-reported disability questionnaire was found to be valid and to possess good psychometric properties. The UDI has a single factor structure and either a 6-item, 7-item or 8-item version can be used to measure disability. For brevity and parsimony, the 6-item UDI is recommended, but further testing of all versions is warranted.”

I have to disagree with the general claim that this conclusion is “not rigorous” given that every component of this conclusion was carefully drawn from the results that are described in detail in the manuscript. Indeed, an attempt was made to be balanced with this conclusion, stating that further work should be done to evaluate the UDI. If Reviewer #3 has a concern with a particular component of the conclusion wording as it currently stands, then please do let me know and I will try to address this particular concern.

Introduction

1) Line 69, page 4: Explain the meaning and role of activities of daily living (ADLs).

Thank you for this suggestion. The sentence being referred to from the introduction is:

“According to the ICF, disability manifests as activity limitations and participation restrictions, thereby placing activities of daily living (ADLs) at the centre of disability assessment.”

ADLs have been a well-established concept in the disability literature since the 1950s, and are commonly used as an indicator of a person’s functional status. I have added a new reference in the existing sentence below that summarises the concept and history of ADLs for the reader’s benefit (line 97): <https://pubmed.ncbi.nlm.nih.gov/29261878/>

“According to the ICF, disability manifests as activity limitations and participation restrictions, thereby placing activities of daily living (ADLs) [6] at the centre of disability assessment.”

2) Line 108-112, page 6: Please describe the innovation of this study.

The lines referred to here are:

“With these considerations in mind, the primary aim of this study was to develop and evaluate the performance of a brief, generic self-reported disability questionnaire. This instrument seeks to address an existing gap in the literature by providing a versatile, yet rigorous, tool for assessing ADL-related disability across various demographic settings and health conditions.”

The justification for this aim is provided in the preceding paragraphs. Namely that: 1) a generic disability measure has several advantages over disease-specific measures; 2) there are few, if any, brief generic self-reported disability questionnaires in existence (instead, most existing generic disability measures rely on third-party in-person observation); 3) those few self-reported measures that do exist have issues such as (lines 105-108) “a focus on a specific aspect of disability, such as task independence [19], they incorporate complex ‘instrumental’ activities that may not be universally relevant or may be outdated [11, 19, 34], or they are very lengthy [11]”.

I have added the following sentence (lines 84-85) to clarify this further for readers:

“Despite this, most generic measures of disability are not self-reported and instead rely on in-person observation.”

Results

Table 1: The gender ratio was 2:1 so the sample was unevenly distributed.

There is little that one can (or should) do regarding the distribution of demographic features (age, sex, etc.) in a general population survey such as the one reported here. Since the analysis was largely descriptive, with no intervention (i.e. a clinical trial), such an ‘imbalance’ does not present a problem. Indeed, this female:male 2:1 ratio is typical of surveys conducted in the United Kingdom (UK), where the research was conducted, and examples of other large UK surveys with similar demographics were cited in the discussion to demonstrate this point. Similar ratios are also seen outside of the UK. The overall sample size (n=402) was sufficient for the purposes of the study. However, as pointed out in the discussion (lines 715-718), “future work should … facilitate the recruitment of under-represented groups to ensure the UDI items are appropriate and meaningful in these groups.”

Discussion

Overall:

The discussion section should be more specific about future research directions, including possible research questions, methods and areas of application.

A comprehensive description of what can be done with a generic disability questionnaire (which is a very broad topic) is beyond the scope of the paper, which is focused on describing the development of the questionnaire itself. In the short-term, the reliability and validity of the questionnaire needs further evaluation before it can be used in epidemiological studies and clinical trials, which is the long-term aim. Hence, several suggestions for future evaluation work are already provided in the discussion section, which should be sufficient:

713-717: “Hence, further studies will be required to create and test any translations of the UDI. Likewise, those with visual impairments would need the questions to be presented via a different medium (e.g., audio or braille) to provide a response. Hence, future work should look at different formats and facilitate the recruitment of under-represented groups to ensure the UDI items are appropriate and meaningful in these groups.”

733-734: “In future studies, this will need to be addressed by testing the UDI with individuals with a higher prevalence of more severe disability.”

Lines 738-740: “In future studies, this bias can be removed by the use of random item ordering, which is relatively straightforward to implement in online surveys.”

Lines 745-746: “Reliability studies should be a priority for future work.”

Many thanks to Reviewer #3 for their time and effort.
